# Quantitative quality control of 3D water tank using image analysis

**DOI:** 10.1002/acm2.70119

**Published:** 2025-05-19

**Authors:** Yuki Tanimoto, Kohei Sugimoto, Kazunobu Koshi, Akira Hiroshige, Shohei Yoshida, Yoshiki Fujita, Atsuki Nakahira, Daiki Nakanishi, Hirofumi Honda, Masataka Oita

**Affiliations:** ^1^ Department of Radiology NHO Kure Medical Center and Chugoku Cancer Center Kure Hiroshima Japan; ^2^ Department of Radiological Technology Faculty of Health Science and Technology Kawasaki University of Medical Welfare Kurashiki Okayama Japan; ^3^ Department of Radiology NHO Fukuyama Medical Center Fukuyama Hiroshima Japan; ^4^ Department of Radiology NHO Shikoku Cancer Center Matsuyama Ehime Japan; ^5^ Division of Radiology Department of Medical Technology Kyushu University Hospital Fukuoka Fukuoka Japan; ^6^ Graduate School of Interdisciplinary Science and Engineering in Health Systems Okayama University Okayama Japan; ^7^ Department of Radiological Technology Ehime University Hospital Toon Ehime Japan; ^8^ Department of Healthcare Science Faculty of Interdisciplinary Science and Engineering in Health Systems Okayama University Okayama Japan

**Keywords:** 3D water tank, drive speed stability, quality control, radiation isocenter, x‐ray image analysis

## Abstract

**Background and objective:**

Accurate beam data acquisition using three‐dimensional (3D) water tanks is essential for beam commissioning and quality control (QC) in clinical radiation therapy. This study introduces a novel method for quantitative QC of the system, utilizing MV images and webcam videos. The stability of the motor drive speed and the positional accuracy of the fixture were evaluated under two measurement modes: “continuous mode” and “step‐by‐step mode.”

**Methods:**

A TRUFIX mounting system (PTW Freiburg Inc., Germany) was used to attach the center of the steel ball to its top, ensuring alignment with the water surface of the tank. To assess deviations from the radiation isocenter, MV images were acquired and compared with digitally reconstructed radiographs (DRRs). These evaluations were performed at different speed settings (slow, medium, and fast) using ET CT Body Marker (BRAINLAB Inc., USA) mounted on the drive unit. A webcam was utilized to capture the images, and custom‐developed tracking software was employed to analyze deviations in driving speed and positional errors.

**Results:**

The mean error of the radiation isocenter was 0.37 ± 0.09 mm. As the motor drive speed increased, the discrepancy between the set speed and the actual speed observed in the analysis also became larger. In “continuous mode,” the deviation from the displayed value was greater than that observed in “step‐by‐step mode.”

**Conclusion:**

It is demonstrated that the proposed analysis method can quantitatively evaluate radiation isocenter misalignment, tank setup position deviation, and both the indicated drive speed values and their stability. At higher drive speeds, the “step‐by‐step mode” showed smaller deviations from the indicated values.

## 1 INTRODUCTION

Accurate acquisition of beam data using three‐dimensional (3D) water tanks is crucial for the registration of beam data in radiotherapy treatment planning systems (RTPS) and for subsequent quality control, both of which are critical in modern high‐precision radiotherapy. Numerous studies have examined the influence of measurement conditions on beam data accuracy and have proposed quality control methods to ensure precise 3D water tank dosimetry.^1^, ^2^, ^3^, ^4^, ^5^ The effects of changes in measurement conditions are also discussed in AAPM Task Group No. 106 (TG‐106), which highlights that the tilt of the scan arm significantly impacts the symmetry of the beam profile, while increasing the scan speed results in higher noise and introduces statistical variations.^6^ Mellenberg et al. emphasized that for the quality control of a 3D water tank, it is essential to assess mechanical alignment, movement speed, and reproducibility using plumb bobs.^7^ However, the methods described in the report are primarily based on visual operational checks. Consequently, they are insufficient for evaluating changes in variability or shifts in baseline conditions since the initial commissioning. Venselaar et al. reported that the reproducibility of relative depth dose data measurements in scanning dosimetry should be within 0.5%, or at least within 1% of the local dose.^8^ Therefore, rigorous and detailed quality assurance is essential for a 3D water tank, as it demands high measurement accuracy.

The 3D water tank can be measured using a “continuous mode,” where the delay time (defined as the time required to move between adjacent measurement points) is optimized to maintain a constant measurement speed. In contrast, in “step‐by‐step mode,” the detector pauses at each measurement point for a specified duration to perform the measurement. To our knowledge, no previous studies have compared these two methods in terms of drive‐speed stability.

Therefore, this study aimed to (1) develop a method for quality control of quantitative 3D water tank measurements using image analysis of x‐ray images or webcam‐captured videos, and (2) evaluate drive speed stability by comparing various measurement modes.

## 2 MATERIALS AND METHODS

### 2.1 Materials

The 3D water tanks and linear accelerators used in this study were BEAMSCAN (PTW Fruiburg Inc., Germany) and TrueBeam STx (Varian Medical Systems Inc., USA), respectively. The 3D water tanks feature an “auto set‐up” procedure that uses a metal water level sensor to measure the tank's water level and assesses the beam profile to correct rotational errors between the radiation isocenter and the water tank.

### 2.2 Displacement between radiation isocenter and mechanical isocenter

**FIGURE 1:**
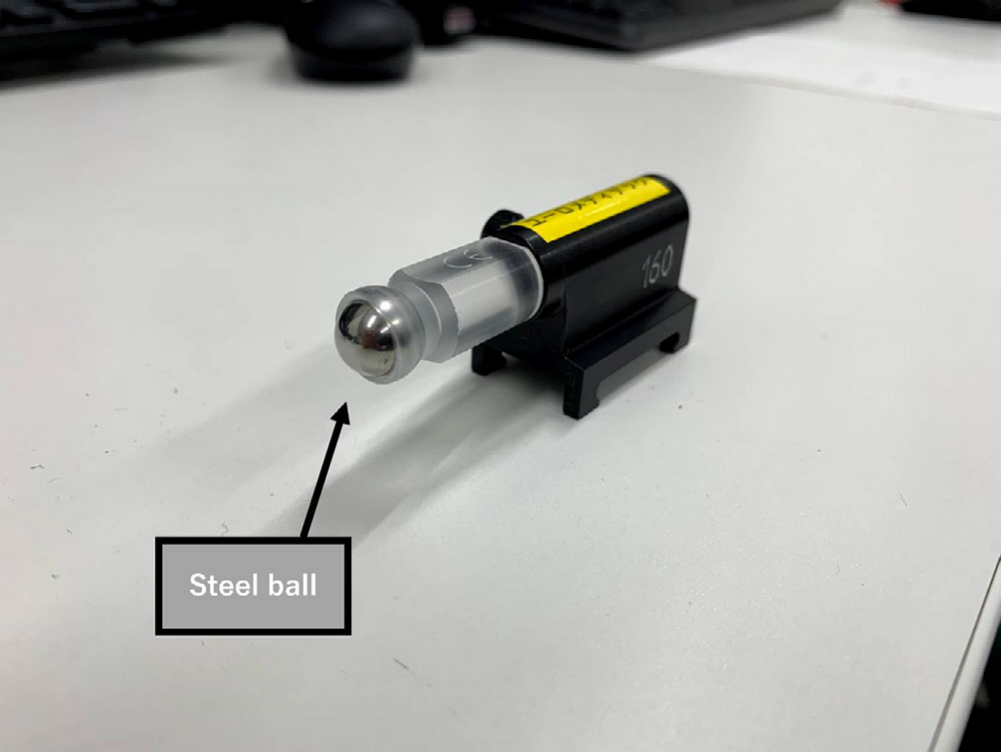
TRUFIX steel ball with a Φ10 mm steel ball affixed to its tip.

**FIGURE 2:**
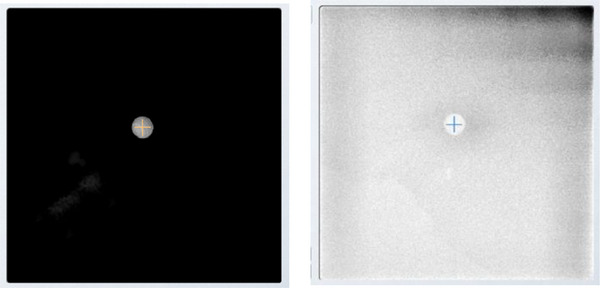
(a) DRR of the TRUFIX steel ball captured with CT before the scan; (b) TRUFIX steel ball imaged with ExacTrac.

After the “auto set‐up” procedure, the TRUFIX steel ball (PTW Freiburg, Germany) was attached to the tip of the steel ball to position its center on the water surface (Figure 1). The TRUFIX steel ball is a 10‐mm‐diameter passivated stainless steel ball, that serves as a reference point during the setup of the Magnetic Resonance Imaging‐compatible BEAMSCAN. Radiographic images were then captured using ExacTrac (Brainlab). Displacement of the ball's center in the radiographic images was assessed by comparing them with digitally reconstructed radiographs (DRRs) (Figure 2). Measurements were taken once a day and repeated 10 times.

### 2.3 Error between indicated and measured drive speed

The ET CT Body Marker (BRAINLAB Inc., USA) is attached to the drive unit and operates in a mode designed to measure the percentage depth dose (PDD) and off‐center ratio (OCR) at an energy level of 10 MV and a field size of 30 × 30 cm^2^.

BEAMSCAN operates in two modes: “continuous mode,” where it is continuously driven and measured, and “step‐by‐step mode,” where it is repeatedly stopped, driven, and measured for a specified period.^9^ The measurements were taken at low, medium, and high speeds (2.00, 5.00, 10.00 mm/s) and (1.50, 1.00, 0.50 s/step) using a Brio C1000s webcam (Logicool, Morges, Switzerland,) at full high definition (1920 × 1080 pixels) at 30 fps.

**FIGURE 3:**
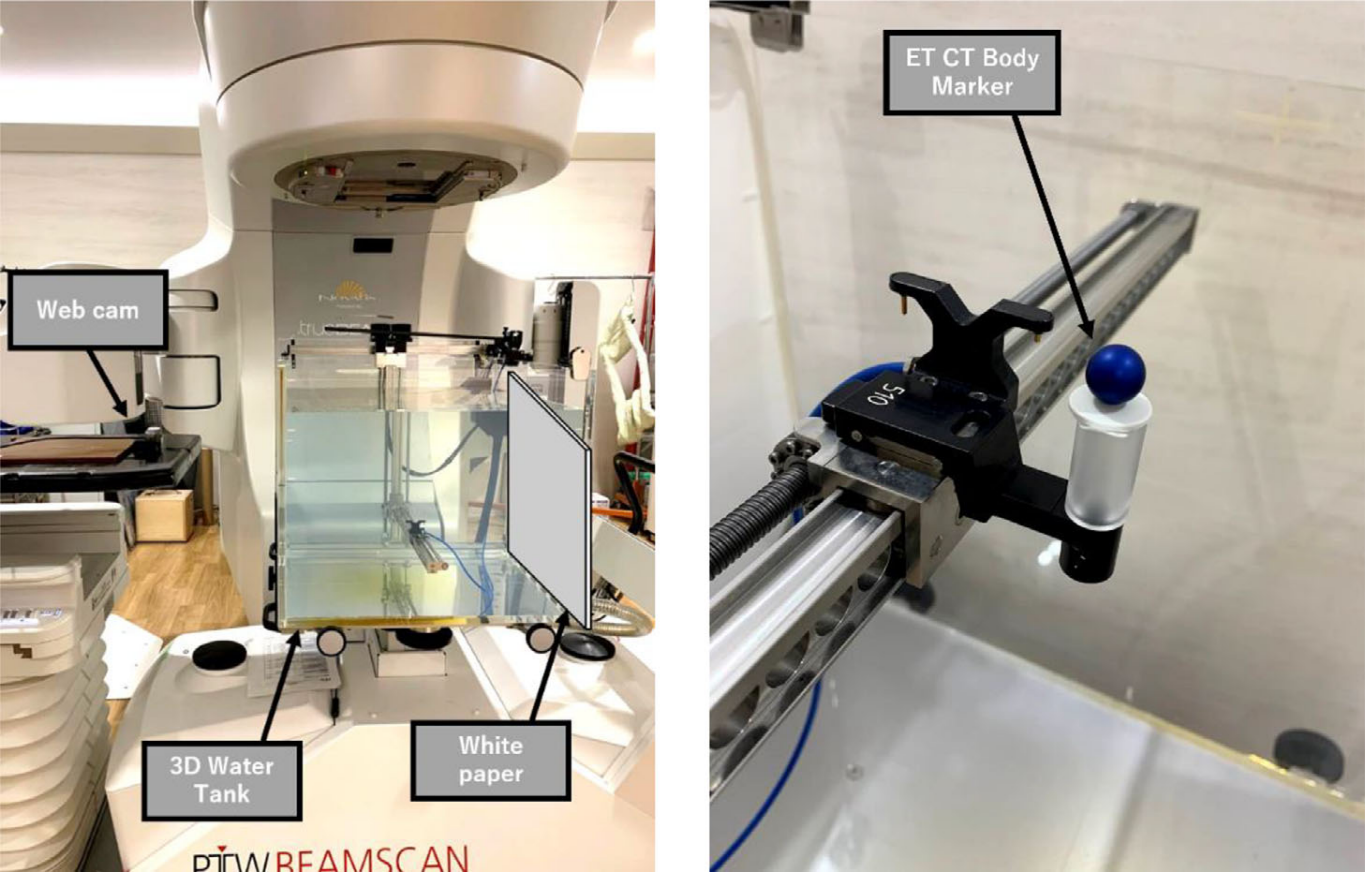
(a) Experimental setup for measuring the error between the indicated and measured drive speeds. (b) ET CT Body Marker attached to the drive unit.

The webcam was placed on the treatment couch. Then, to ensure consistent distance from the ET CT Body Marker each time, ruled lines were drawn on the webcam to align with the cross‐hair line in the left‐right (LR), superior‐inferior (SI), and anterior‐posterior (AP) directions. The webcam was then moved ‐45 cm in the SI direction and ‐15 cm in the AP direction (Figure 3).

**FIGURE 4:**
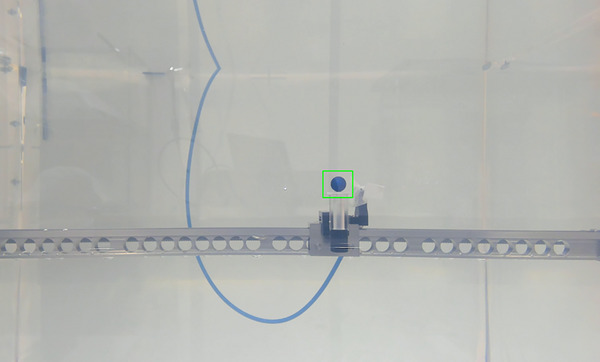
Measurement of the driving speed of the ET CT Body Marker using tracking software.

Speed validation was conducted using Microsoft Visual Studio Code and OpenCV (version 4.6.0.66). A tracking software based on the Kernelized Correlation Filter method was developed to extract time‐series position coordinates^10^, ^11^ (Figure 4). In “continuous mode,” the slope of the position versus time graph was defined as the drive speed, while in “step‐by‐step mode,” the average stop time at each point was calculated. The videos were recorded once per day for a total of 10 days, and each video was analyzed three times to ensure the accuracy of the tracking software.

## 3 RESULTS

### 3.1 Displacement between radiation isocenter and mechanical isocenter

**FIGURE 5:**
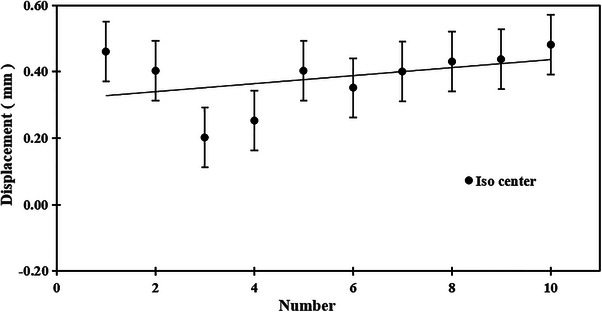
Displacement of the TRUFIX steel ball at the radiation isocenter. The error bars indicate the standard deviation of 10 measurements.

The average displacement from the radiation isocenter was 0.37 ± 0.09 mm, with a maximum value of 0.48 mm and a minimum value of 0.20 mm (Figure 5).

### 3.2 Error between indicated and measured drive speed

**FIGURE 6:**
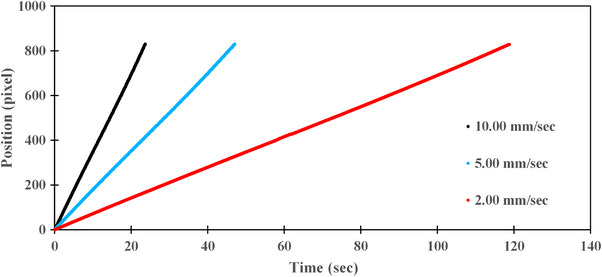
Position of the ET CT Body Marker during PDD measurement in “continuous mode,” with drive speeds adjusted to low, medium, and high.

**FIGURE 7:**
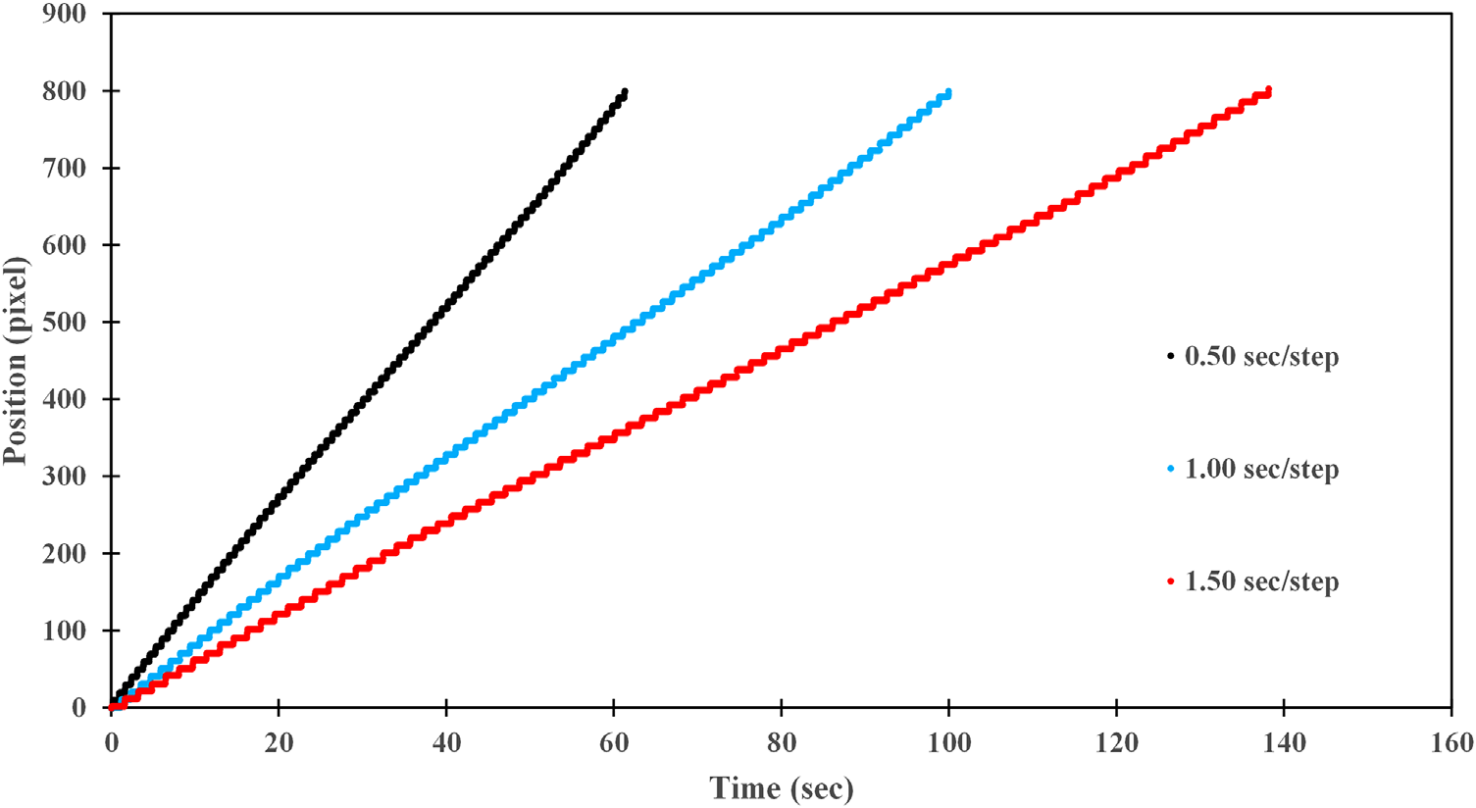
The positions of ET and CT body markers during PDD measurements conducted in “step‐by‐step mode” at varying drive speeds (low, medium, and high).

Figure 6 illustrates the time‐dependent changes in position coordinates at different speeds when measured in “continuous mode” using the Kernelized Correlation Filter method. Figure 7 depicts the changes in position coordinates measured in “step‐by‐step mode.”

The average drive speed in “continuous mode” was 2.07 (SD = 0.00) mm/s, 5.19 (SD = 0.01) mm/s, and 10.38 (SD = 0.01) mm/s, corresponding to 2.0, 5.0, and 10.0 mm/s, respectively. In “step‐by‐step mode,” the average speeds were 1.53 (SD = 0.01) s/step, 1.08 (SD = 0.01) s/step, and 0.65 (SD = 0.01) s/step, corresponding to time intervals of 1.5, 1.0, and 0.5 s/step, respectively. For OCR, the average speeds in “continuous mode” were 2.02 (SD = 0.01) mm/s, 5.11 (SD = 0.02) mm/s, and 10.21 (SD = 0.03) mm/s, while in “step‐by‐step mode,” the speeds were 1.53 (SD = 0.01) s/step, 1.08 (SD = 0.01) s/step, and 0.64 (SD = 0.01) s/step (Tables 1 and 2).

**TABLE 1:** The error between the measured and indicated drive speeds in “continuous mode.”

Number	PDD			OCR		
	2.00 (mm/s)	5.00 (mm/s)	10.00 (mm/s)	2.00 (mm/s)	5.00 (mm/s)	10.00 (mm/s)
1	2.06 (0.00)	5.20 (0.00)	10.40 (0.00)	2.02 (0.00)	5.08 (0.00)	10.19 (0.01)
2	2.06 (0.01)	5.20 (0.00)	10.38 (0.00)	2.01 (0.00)	5.09 (0.01)	10.20 (0.00)
3	2.06 (0.00)	5.19 (0.00)	10.32 (0.01)	2.03 (0.00)	5.12 (0.00)	10.25 (0.05)
4	2.07 (0.00)	5.18 (0.03)	10.37 (0.01)	2.01 (0.00)	5.07 (0.00)	10.14 (0.03)
5	2.07 (0.01)	5.21 (0.00)	10.37 (0.01)	1.99 (0.03)	5.07 (0.00)	10.16 (0.02)
6	2.07 (0.01)	5.20 (0.01)	10.38 (0.02)	2.00 (0.01)	5.15 (0.04)	10.30 (0.09)
7	2.07 (0.00)	5.22 (0.00)	10.40 (0.01)	2.05 (0.05)	5.10 (0.00)	10.21 (0.01)
8	2.07 (0.00)	5.07 (0.01)	10.37 (0.01)	2.01 (0.00)	5.16 (0.15)	10.17 (0.02)
9	2.07 (0.00)	5.21 (0.00)	10.39 (0.00)	2.03 (0.00)	5.13 (0.00)	10.21 (0.05)
10	2.07 (0.00)	5.22 (0.01)	10.41 (0.01)	2.01 (0.04)	5.08 (0.04)	10.25 (0.02)
Mean	2.07 (0.00)	5.19 (0.01)	10.38 (0.01)	2.02 (0.01)	5.11 (0.02)	10.21 (0.03)
Error from indicated value	+0.07	+0.19	+0.38	+0.02	+0.11	+0.21

*Note*: Value represents the mean (standard deviation). Drive speeds were measured under measuring mode conditions (PDD and OCR) at varying speed levels: Low, medium, and high.

**TABLE 2:** The error between the measured and indicated drive speeds in “step‐by‐step mode.”

Number	PDD			OCR		
	1.50 (s/step)	1.00 (s/step)	0.50 (s/step)	1.50 (s/step)	1.00 (s/step)	0.50 (s/step)
1	1.53 (0.00)	1.07 (0.00)	0.62 (0.00)	1.56 (0.00)	1.08 (0.00)	0.66 (0.00)
2	1.54 (0.02)	1.11 (0.00)	0.65 (0.02)	1.53 (0.00)	1.10 (0.02)	0.64 (0.02)
3	1.56 (0.01)	1.11 (0.00)	0.64 (0.01)	1.55 (0.01)	1.09 (0.01)	0.63 (0.00)
4	1.53 (0.00)	1.10 (0.01)	0.65 (0.00)	1.56 (0.00)	1.08 (0.02)	0.66 (0.02)
5	1.54 (0.01)	1.02 (0.01)	0.67 (0.01)	1.53 (0.00)	1.05 (0.00)	0.60 (0.03)
6	1.53 (0.02)	1.08 (0.01)	0.64 (0.02)	1.54 (0.02)	1.14 (0.02)	0.67 (0.02)
7	1.52 (0.03)	1.07 (0.00)	0.63 (0.00)	1.56 (0.01)	1.07 (0.02)	0.65 (0.00)
8	1.52 (0.00)	1.11 (0.01)	0.66 (0.01)	1.54 (0.01)	1.11 (0.01)	0.72 (0.00)
9	1.54 (0.01)	1.08 (0.02)	0.60 (0.02)	1.45 (0.01)	1.01 (0.00)	0.65 (0.00)
10	1.50 (0.03)	1.01 (0.00)	0.58 (0.01)	1.52 (0.02)	1.06 (0.02)	0.59 (0.02)
Mean	1.53 (0.01)	1.08 (0.01)	0.64 (0.01)	1.53 (0.01)	1.08 (0.01)	0.65 (0.01)
Error from indicated value	+0.03	+0.08	+0.14	+0.03	+0.08	+0.15

*Note*: Value represents the mean (standard deviation). Drive speeds were measured under measuring mode conditions (PDD and OCR) at varying speed levels: Low, medium, and high.

As the drive speed increased or the stop time decreased, the deviation from the indicated value became more pronounced. Repeated analyses using the tracking software had minimal impact on the results. The error observed in “continuous mode” was larger than that in “step‐by‐step mode.”

## 4 DISCUSSION

### 4.1 Displacement between radiation isocenter and mechanical isocenter

The Winston‐Lutz test has long been used to accurately determine the radiation isocenter of a linear accelerator in radiation therapy.^12^ Weiliang et al. investigated this to quantify the alignment between the electronic portal imaging device (EPID) and the radiation isocenter, reporting a deviation of 0.82 mm at a gantry angle of 180°, compared to the reference angle of 0°.^13^ By using a TRUFIX steel ball and capturing images from multiple directions with Exactrac, this method achieved a high detection accuracy, averaging 0.37 ± 0.09 mm/s similar to the Winston‐Lutz test, it was found to enable three‐dimensional analysis. Furthermore, the method is straightforward, requiring only the replacement of the chamber with a TRUFIX steel ball, and the entire process can be completed within a few minutes.

### 4.2 Error between indicated and measured drive speed

The graphs in Figures 6 and 7 and the various average values in Tables 1 and 2 indicate that the tracking software effectively captured the drive speed measurements.

Mohammad et al. demonstrated that surface waves generated by the motion of the scanning arm influence measurement errors, with the impact becoming more significant at higher scanning speeds. They also found that accurate measurements can be achieved at speeds ranging from 1.0 to 5.0 mm/s.^14^ The discrepancy between the indicated and measured values was attributed to changes in the stability of the drive speed. Additionally, since the images were captured in a water‐filled tank, the refraction of the moving images caused by the water was also considered a contributing factor. However, the deviation increased with higher speeds, while at lower speeds, it was nearly identical to the indicated values. This suggests that the primary cause of the discrepancy was the instability of the drive speed. TG‐106 states that for small ionization chambers with low signal output, reducing the drive speed is necessary to increase signal strength and reduce statistical fluctuations.^6^ The findings of this study also highlight the importance of selecting an appropriate drive speed from the perspective of drive speed stability as well.

### 4.3 Comparison of measurement modes

When comparing the “continuous mode” and “step‐by‐step mode” the error relative to the indicated value was larger in “continuous mode.” Although the maximum error was only +0.38 mm/s, it was evident that speed stability was higher in “step‐by‐step mode,” particularly at high speeds.

The “continuous mode” exhibited larger errors compared to the indicated values. Although the maximum error was only +0.38 mm/s, “step‐by‐step mode” demonstrated high speed stability, particularly at high speeds. On the other hand, at low and medium speeds, the accuracy of “continuous mode” and “step‐by‐step mode” mode was found to be nearly identical. This result suggests that under specific speed conditions, either mode can be selected without causing significant differences in measurement accuracy.

The “continuous mode” has the advantage of reducing measurement time; however, maintaining speed stability at high speed can be challenging. Conversely, “step‐by‐step mode” provides superior stability at high speed but tends to require longer measurement times. Based on the findings of this study, it is concluded that choosing the appropriate mode based on the measurement objective can enable both efficient and accurate measurements.

### 4.4 Tolerance evaluation of isocenter displacement and drive speed errors

AAPM TG‐142 recommends that the displacement between the mechanical isocenter and the radiation isocenter be kept within 1 mm.^15^ Additionally, AAPM TG‐53 specifies that the accuracy of beam positioning should be within 1 mm.^16^ Therefore, it is reasonable to consider a displacement threshold of 1 mm as an acceptable limit in this study. Meeting this criterion ensures positional accuracy in radiation therapy, thereby contributing to the maintenance of treatment precision.

The impact of drive speed on the profile depends on the type of ionization chamber used; thus, this study does not provide specific tolerance values from the perspective of profile influence. However, for long‐term quality assurance, the use of confidence limits is considered appropriate.^8^, ^17^ By applying confidence limits, errors can be assumed to fall within ± 1.96 standard deviations (SD) with 95% probability, enabling high‐accuracy management. Consequently, an evaluation based on confidence limits is recommended. The confidence limit is given by Equation (1):

(1) $$Confidence\;limit=\left|Mean\right|+1.96\times SD$$

### 4.5 Clinical implementation

By performing QA using this method before commissioning a radiation therapy device, uncertainties in the water tank's performance can be identified early, allowing for necessary adjustments and improvements. This enhances measurement accuracy during commissioning and ensures more reliable results.

Furthermore, this method is not limited to commissioning but can also be incorporated into existing QA programs for continuous accuracy management. Regular evaluations help ensure the long‐term reliability of the treatment device and maintain measurement precision.

### 4.6 Limitations of the study

The limitations of this study include the fact that only short‐term validation was performed. Further research is necessary to confirm long‐term stability. Additionally, since the vendor did not disclose detailed information, such as allowable thresholds, it is deemed important to establish a baseline using the proposed method and to compare and verify long‐term variations.

## 5 CONCLUSION

We proposed a method for quantitatively analyzing a 3D water tank using image analysis. The results demonstrated that the proposed method can accurately evaluate the deviation between the radiation isocenter and the tank position, as well as the indicated values and stability of the drive speed. When the drive speed is high, the “step‐by‐step mode” exhibits greater stability in terms of speed consistency.

## AUTHOR CONTRIBUTIONS

Yuki Tanimoto: Conceptualization and study design, software development, collected data, and wrote the article. Kohei Sugimoto: Software development and review. Kazunobu Koshi, Akira Hiroshige, Shohei Yoshida, Yoshiki Fujita, Atsuki Nakahira, Daiki Nakanishi: Investigation. Hirofumi Honda: Study design and review. Masataka Oita: Conceptualization, methodology, and writing–review and editing. All authors read and approved the final manuscript.

## CONFLICT OF INTEREST STATEMENT

The authors declare no conflicts of interest.

## References

[1] Tanaka Y , Mizuno H , Akino Y , Isono M , Masai N , Yamamoto T . Do the representative beam data for TrueBeam™ linear accelerators represent average data? J Appl Clin Med Phys. 2019;20(2):51‐62. doi:10.1002/acm2.12518 PMC6370991 30636358

[2] Beddar AS , Mason DJ , O'Brien PF . Absorbed dose perturbation caused by diodes for small field photon dosimetry. Med Phys. 1994;21(7):1075‐1079. doi: 10.1118/1.597350 7968839

[3] Akino Y , Fujiwara M , Okamura K , et al. Characterization of a microSilicon diode detector for small‐field photon beam dosimetry. J Radiat Res. 2020;61(3):410‐418. doi: 10.1093/jrr/rraa010 32211851 PMC7299273

[4] IAEA . Absorbed dose determination in external beam radiotherapy an international code of practice for dosimetry based on standards of absorbed dose to water. Tech Rep Series. 2000;398:1–201.

[5] Chang Z , Wu Q , Adamson J , et al. Commissioning and dosimetric characteristics of True Beam system: composite data of three TrueBeam machines. Med Phys. 2012;39(11):6981‐7018. doi: 10.1118/1.4762682 23127092

[6] Das IJ , Cheng CW , Watts RJ , et al. Accelerator beam data commissioning equipment and procedures: report of the TG‐106 of the Therapy Physics Committee of the AAPM. Med Phys. 2008;35(9):4186‐4215. doi: 10.1118/1.2969070 18841871

[7] Mellenberg DE , Dahl RA , Blackwell CR . Acceptance testing of an automated scanning water phantom. Med Phys.1990;17(2):311‐314. doi: 10.1118/1.596510 2333057

[8] Venselaar J , Welleweerd H , Mijnheer B . Tolerances for the accuracy of photon beam dose calculations of treatment planning systems. Radiother Oncol. 2001;60(2):191‐201. doi: 10.1016/s0167-8140(01)00377-2 11439214

[9] Kafi MAA , Mwidu U , Moftah B . Continuous versus step‐by‐step scanning mode of a novel 3D scanner for CyberKnife measurements. Appl Radiat Isot. 2015;105:88‐91. doi: 10.1016/j.apradiso.2015.07.020 26265091

[10] OpenCV . Accessed May 10, 2023. https://opencv.org/

[11] Henriques JF , Caseiro R , Martins P , Batista J . High‐speed tracking with kernelized correlation filters. IEEE. 2015;37(3):583‐596. doi: 10.1109/TPAMI.2014.2345390 26353263

[12] Lutz W , Winston KR , Maleki N . A system for stereotactic radiosurgery with a linear accelerator. Int J Radiat Oncol Biol Phys. 1988;14(2):373‐381. doi: 10.1016/0360-3016(88)90446-4 3276655

[13] Du W , Yang J , Luo D , Martel M . A simple method to quantify the coincidence between portal image graticules and radiation field centers or radiation isocenter. Med Phys. 2010;37(5):2256‐2263. doi: 10.1118/1.3397452 20527559

[14] Bakhtiari M . Effect of surface waves on radiotherapy dosimetric measurements in water tanks. J Med Phys. 2011;36(4):230‐233. doi: 10.4103/0971-6203.89972 22228932 PMC3249734

[15] Klein EE , Hanley J , Bayouth J , et al. Task Group 142 report: quality assurance of medical accelerators. Med Phys. 2009;36(9):4197‐4212. doi: 10.1118/1.3190392 19810494

[16] Fraass B , Doppke K , Hunt M , et al. American Association of Physicists in Medicine Radiation Therapy Committee Task Group 53: quality assurance for clinical radiotherapy treatment planning. Med Phys. 1998;25(10):1773‐1829. doi: 10.1118/1.598373 9800687

[17] Palta JR , Kim S , Li JG , Liu C . Intensity‐Modulated Radiation Therapy, The State of the Art. Medical Physics Publishing. 2003: 593–612.

